# The impact of land transfer policy on sustainable agricultural development in China

**DOI:** 10.1038/s41598-024-57284-8

**Published:** 2024-03-25

**Authors:** Congjia Huo, Lingming Chen

**Affiliations:** 1 Department of Economics, Business School (School of Quality Management and Standardization), Foshan University, Foshan, 528225 Guangdong China; 2 Research Centre for Innovation & Economic Transformation, Foshan University, Foshan, Guangdong China; 3 Department of Economic Statistics, School of Economics and Management, Xinyu University (XYU), Xinyu, 338004 Jiangxi China

**Keywords:** Continuous DID, Land transfers, Land transfer policies, Agricultural sustainability, China, Environmental sciences, Environmental social sciences

## Abstract

At present, China’s agricultural development presents problems such as increasingly tight constraints on water and soil resources, increased pollution in the process of agricultural production, and noticeable degradation of agricultural ecosystems. So, it is urgent to promote the sustainable development of agriculture. From the perspective of land transfer policy, based on the panel data of 30 regions in China from 2006 to 2020, this paper uses the entropy weight method to calculate the level of sustainable agricultural development. Based on the analysis of the impact mechanism of land transfer policy on sustainable agricultural development, the relationship between land transfer policy and sustainable agricultural development is empirically tested by the continuous difference-in-difference method. The study found that the overall level of sustainable agricultural development in China is relatively low but shows an upward trend. The land transfer policy significantly promoted the sustainable development of agriculture in China. This conclusion is still valid after a series of validity tests and robustness tests. Finally, the corresponding policy suggestions are put forward according to the theoretical analysis and empirical results. Future research will focus on indicators challenging to quantify in agricultural sustainable development and find effective methods to incorporate them into the indicator system. At the same time, find the data of Prefecture-level cities in major grain-producing areas and further improve the measurement and research of agricultural sustainable development.

## Introduction

Agriculture is the foundation of the state, and the healthy and stable development of agriculture is the basis for the rapid development of the national economy of a country or region. For China, agriculture is the bedrock of its national economy. The quality and efficiency of agricultural production are related to the country’s food security and economic development. However, China’s agricultural production has faced practical problems such as limited land, stretched water resources, and excessive use of fertilizers and pesticides. Currently, China’s agricultural development shows increasingly tight constraints on water and soil resources, increased pollution in agricultural production, and noticeable degradation of agricultural ecosystems. At the same time, China’s water and soil resources management, ecological compensation, and other systems and mechanisms are not perfect, and the traditional mode of agricultural development has made it difficult to solve the problems caused by the acceleration of modernization. Therefore, we must adopt a sustainable agrarian production model to ensure long-term stable agricultural production and sustainable agricultural development in China. In recent years, with the increasing consensus on sustainable agricultural development, China’s sustainable rural development has achieved good results in four aspects: the sustained growth of total farm production capacity and farmers’ income, the steady improvement of the utilization level of agricultural resources, the continuous increase of agricultural ecological protection and construction, and the gradual improvement of rural living environment.

China’s agricultural production model is dominated by a small-scale peasant economy, showing the characteristics of scattered land, low comprehensive management capacity of farmers’ families, backward agricultural production methods, and extensive production and sales of farm products. In addition, the phenomenon of land fragmentation and decentralization will hinder the sustainable development of agriculture. Land transfer policy aims to promote agricultural modernization and large-scale operation, improve agricultural production and economic efficiency, and thus promote sustainable agricultural development. Zhongfa [2010] No.1, issued by the Chinese government in 2010, clearly stated that “we will announce the reform of the rural land management system in an orderly manner and accelerate the registration and certification of rural collective land ownership, homestead use rights, and joint construction land use rights” The No. 1 document of 2010 proposed five measures to coordinate urban and rural development. The government has taken multiple measures to improve the social security and public service levels of mobile populations and vulnerable groups and actively promoted rural land transfer. Specifically reflected in the following two aspects: On the one hand, improving the land transfer market: The document proposes to strengthen the management and service of land contract management rights transfer, which means establishing and improving the land transfer market mechanism to ensure the transparency and efficiency of land transfer.

**Figure 1:**
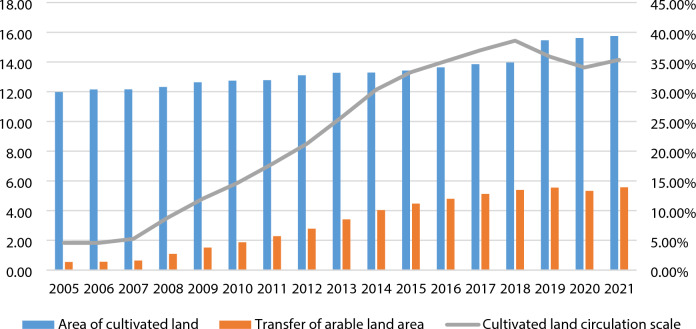
Cultivated land area and transferred cultivated land area in China from 2005 to 2021, and land transfer scale (unit: 100 million mu). *Note* Land transfer scale = total area of cultivated land transferred/developed land area * 100%. The left Y-axis shows the scale of land transfer (unit: 100 million acres), while the right Y-axis shows the scale of land transfer (unit:%).

On the other hand, protecting the rights and interests of farmers: The document mentions the strict implementation of the Rural Land Contract Management Dispute Mediation and Arbitration Law, which is to protect the rights and interests of farmers in land contract management and prevent infringement during the land transfer process. With the continuous revision and improvement of the land transfer policy, the scale of land transfer in China has increased rapidly. Figure 1 shows the land area and cultivated land area transferred in China from 2005 to 2021, as well as the scale of land transfer.

According to Fig. 1, the scale of land transfer in China has increased significantly. In 2007, the scale of land transfer was only 5.24%, and by 2018, the value increased to 38.59%. It is difficult for cultivated land resources to grow. The total area of cultivated land in China has changed slightly, and the area of cultivated land has increased yearly. In 2016, China’s “Three Rights Separation” policy gave the due legal status of land management rights. After that, the scale of land transfer showed a rapid growth model and tended to stabilize. The land transfer policy can theoretically promote sustainable agricultural development. From the data point of view, the trend of the two is the same, so is there a driving force for sustainable agricultural development brought about by land transfer policy? How much does the land transfer policy affect the sustainable development of agriculture? How can we further promote sustainable agricultural development through a land transfer policy? This paper uses the continuous difference-in-difference (DID) method to verify whether the transfer policy can promote the sustainable development of agriculture based on calculating the sustainable development level in 30 regions of China.

The rest of this paper is organized as follows: Section “Literature review and theoretical mechanism” is a literature review and theoretical hypothesis on related topics; In Section “Index system and construction of agricultural sustainable development level”, the panel data of 30 regions in China from 2006 to 2020 are used to calculate the sustainable development level of regional agriculture by entropy weight method; In Section “Empirical study on the impact of land process policy on the level of sustainable agricultural development”, the continuous DID model is set up, and the variable description and data source are given. At the same time, it empirically tests the impact of land transfer policy on sustainable agricultural development and tests the effectiveness and robustness of the model. Section “Conclusions, policy recommendations, and research prospects” is this paper’s conclusion, policy recommendations, and future research directions.

## Literature review and theoretical mechanism

### Literature review

#### Research on the calculation of sustainable agricultural development

Academic research on sustainable agricultural development mainly includes the optimization of agrarian resource allocation, agricultural productivity, and agricultural trade (Alagh^1^; Wen et al.^2^; Zhou^3^). Calculating sustainable agricultural development is the premise of studying sustainable agricultural development. Many documents calculate the level of sustainable agricultural development in different regions or periods through various methods, mainly using the entropy weight method. Indicators are primarily selected from subsystems such as population, society, economy, environment, and resources to construct an indicator system for sustainable agricultural development for Calculation (Yuan and Qi^4^; Zhao et al.^5^; Zhang et al.^6^). Other scholars have used other methods to estimate sustainable agricultural development: Liu and Ma^7^ lay on the nonlinear and grey character of the agricultural sustainable development system. Using grey system theory and the neural network simulation method, we set up scientific and reasonable calculation and forecast models for the coordination degree of agricultural sustainable development systems. A questionnaire was developed based on the analytical hierarchy process (AHP) (Poursaeed et al.^8^). Ye and Chen^9^ used the DEA analysis method to divide the agricultural sustainable development system into four subsystems: survival security, agricultural economic development, rural social progress, and agricultural ecological protection. Miao et al.^10^ used a weighted comprehensive evaluation, coordination, and development obstacle models to analyze the current situation, potential, and obstacle factors of sustainable agricultural development in the study area. Tang and Liu^11^ constructed the evaluation index system of agricultural sustainable development level from five aspects: population, society, economy, environment, and resources. They used the coupling coordination model to analyze the coupling degree and coordination degree among the subsystems of sustainable agricultural development in the Yangtze River Economic Zone. Adamopoulos et al.^12^ used panel data and quantitative frameworks from China’s household level to record the degree and consequences of agricultural factor mismatch. They found that improper resource allocation among farmers can reduce agricultural production efficiency. Adenle et al.^13^ used evidence of the benefits and challenges associated with sustainable farming practices in Africa. They found that improving agricultural sustainability is crucial for food security and poverty reduction, especially for achieving sustainable development goals by 2030. However, agricultural sustainability alone cannot solve all of these issues.

## Research on land transfer policy and sustainable agricultural development

Many factors affect the sustainable development of agriculture, including natural, economic, social, and technological obstacles, among which the insufficient supply of agricultural systems, primarily agrarian land systems, is an essential factor. Under the economic structure dominated by agriculture and the level of traditional technology, agricultural production’s core competitiveness is producers’ enthusiasm. Stable land use rights are the institutional basis for ensuring farmers’ enthusiasm and stimulating sustainable agricultural development (Besley^14^; Chari et al.^15^). To promote farmers’ enthusiasm for farm investment and achieve sustainable land use and agricultural growth, agricultural protection must be carried out to protect the economic interests of farm producers and operators (Pang^16^). However, the imperfect system and mechanism of agricultural land transfer have directly become a severe obstacle to the sustainable development of agriculture (Hu^17^). An imperfect land transfer market will affect the efficiency of agricultural land resource allocation (Rosticceria^18^). Small-scale farmers’ discriminatory land policy, land taxation, input subsidies, imperfect land factor market, and credit constraints are the main reasons for affecting resource allocation and sustainable development in the agricultural sector (Adamopoulos^19^). Adamopoulos^20^ used Philippine household data and found that unreasonable land transfer policies would inhibit the optimal allocation of land resources. Bolhuis et al.^21^ used Indian household data and found that land flow transfer promotes land redistribution, thus improving agricultural productivity. Xia and Zeng^22^ tested the impact of changes in the farmland transfer system on sustainable agricultural development, and, combined with the development status of China’s “three rural” issues and the requirements of sustainable agricultural development, analyzed the way out of the reform of the farmland transfer system in the case of normalization of agricultural land transfer.

Li and Zhu^23^ found that legalizing land transfer and land property reform can increase agricultural investment and improve land quality. Moreover, the benefits of strengthening land quality are unevenly distributed across different socio-economic backgrounds. Fei et al.^24^ used the PSM method to construct a counterfactual framework to analyze the impact of land transfer on agricultural land efficiency. The average land use efficiency in developing countries is relatively low, and provinces that transfer land are more effective in land use than other provinces. Yang et al.^25^ measured the sustainability of farmers’ livelihoods based on field survey data from 650 households in Hubei Province, China. They found that land transfer can significantly improve the sustainability of farmers’ livelihoods, which increases with the increase of land transfer area. Cao et al.^26^ found that the positive impact of land leasing and the negative impact of land fragmentation on agricultural production efficiency indicate that it is essential to promote land consolidation policies by incorporating land parcel connectivity interventions in the land leasing market. The mainstream of land transfer is the spontaneous, slow, and internal transfer in rural areas, which promotes the concentration of land to some farmers. In the process of land transfer and class construction, the form of a middle agricultural economy has emerged, and the rural middle class and its shaped middle agricultural economy provide strong support for the sustainable development and modernization of agriculture (Liu^27^).

Compared with existing literature, the possible contributions of this article are mainly reflected in two aspects. Firstly, this article analyzes how the implementation of land transfer policies affects sustainable agricultural development from the perspective of land transfer policies. At the same time, it provides new empirical evidence for the government to formulate policies to promote sustainable agricultural development. Secondly, this article constructs a small sample continuous double difference model based on random inference. At the same time, it also reduces endogeneity problems caused by selective bias and reverse causality. In addition, the impact of land transfer policies on sustainable agricultural development is also empirically tested.

## Theoretical mechanism

The reform of land transfer policies will promote the increase of land transfer scale in China, thereby improving the efficiency of land resource utilization and agricultural production, protecting the agricultural ecological environment, and promoting sustainable agricultural development through these three paths. Specifically:

1. Land transfer policy can improve the efficiency of land resource use

Farmers’ average household land management area has been too narrow for a long time, seriously restricting agricultural machinery and advanced agricultural technology (Yao and Hamori^28^; Ying et al.^29^). Land use rights cannot be freely transferred, so many young agricultural laborers flow into cities. It is difficult for surplus rural labor to engage in efficient agricultural production activities, and some areas have experienced abandonment. The reform of land transfer policies has dramatically promoted the behavior of agricultural land transfer. Expanding the farm production scale promotes sustainable agricultural development from two aspects.

On the one hand, with the gradual improvement of land transfer policies, families who are unable to engage in agricultural production transfer their land use rights to large farmers, and a large number of scattered and small plots of land gather to form a large-scale and efficient agrarian production model; On the other hand, land property rights reform has promoted land redistribution among farmers, promoting land trusteeship, transfer, and intensive use (Chari et al.^15^). This is beneficial for improving land use efficiency, reducing excessive land reclamation and damage, and protecting rural resources and the environment. Land transfer policies can promote more rational allocation and utilization of land resources, avoid land idleness and waste, improve land use efficiency, and promote sustainable agricultural development.

(2) Land transfer policy can improve the efficiency of agricultural production

The scale of small farmers’ families is relatively small, and the funds and resources of farmers’ families are relatively scarce, so it isn’t easy to afford high scientific and technological investment and equipment procurement. At the same time, due to the lack of professional knowledge and technical skills, farmers often do not have enough ability to carry out technological innovation and application, and it is challenging to meet the requirements of market demand and industrial upgrading. Coupled with the long-term influence of traditional concepts and habits, farmers lack the spirit of pioneering and enterprising and the understanding of technological innovation, which makes it challenging to adapt to the requirements of the scientific and technical era. The land transfer policy has promoted the formation of large-scale agricultural production. With the entry of capital elements into the primary industry and the continuous introduction of advanced technology and equipment in agricultural production, China’s agriculture has gradually achieved large-scale, mechanized, and standardized production. It reduces production costs and improves agricultural production efficiency and market competitiveness. The land transfer policy can promote the centralized allocation of agricultural means of production, improve production efficiency and economic benefits, and thus promote sustainable agricultural development.

(3) Land transfer policy can protect the agricultural ecological environment

Agricultural zoning design has important strategic significance, and China’s previous small-scale and fine agricultural production model is challenging to achieve agricultural layout. The land transfer policy can promote reasonable agricultural layout and planting structure adjustment. From the micro level, optimizing agricultural layout can avoid environmental problems such as soil degradation and soil erosion caused by excessive reclamation and pesticide use and help protect the agricultural ecological environment. From the macro level, according to the local climate, topography, soil and other natural conditions and economic development level, the distribution and utilization direction of agricultural land are determined, and the layout and spatial distribution of agricultural land are rationally planned, which significantly promotes the sustainable development of agriculture.

Through large-scale land management, land transfer accelerates the process of farmers’ non-agriculturalization, inherits urbanization, and starts agricultural modernization, which is the premise of realizing the synchronous and coordinated development of urbanization and agricultural modernization in China (Yang^30^). Industrialization, urbanization, marketization, and agricultural industrialization promote the gradual improvement of land transfer policies, and the rural land property rights system has evolved in the direction of clarifying the subject of property rights, ensuring the right of land transfer and facilitating land transfer (Huang and Xie^31^). To sum up, land transfer is an effective way to promote sustainable agricultural development. It can improve land use efficiency and protect the agricultural ecological environment, promote agrarian modernization, transformation, and upgrading, improve farmers’ income and living standards, and promote rural transformation and development. The land transfer policy has further improved China’s agricultural system, effectively alleviated the problems of significant differences in the quality of farm products, weak competitiveness, and low production efficiency brought about by fine-grained agricultural production, and also solved the problems of insufficient rural labor force and limited production of farm products brought about by the entry of young agricultural labor force into cities. Based on this, this paper proposes two hypotheses to be tested:

This article proposes a research hypothesis: land transfer policy can promote sustainable agricultural development.

## Index system and construction of agricultural sustainable development level

### Index construction and data source

#### Index construction

Sustainable agriculture refers to the formation of sustainable development principles. It combines traditional agriculture with modern technology. And based on protecting natural resources. A new agricultural production method that also achieves ecological balance and economic benefits. Specifically, sustainable agriculture is guided by environmental principles and ecological economic laws. It is simultaneously focusing on managing and protecting natural resources. It combines traditional agriculture with modern technology. It aims to meet everyday people’s and future generations’ needs for agricultural products. The main characteristics of this new agricultural production method are as follows: The production process balances economic benefits with ecological protection. At the same time, the goal is to achieve sustainable, fair, efficient, and diverse agricultural production. Overall, sustainable agricultural models are entities with structures and functions. Integrating ecological, economic, and social elements demonstrates their status and interaction in the system network. This model reflects explicitly the sustainable utilization of resources. The sustainable development of agriculture needs the mutual influence, promotion, and joint development of population, economy, resources, society, environment, and other factors. To scientifically measure the level of regional agricultural sustainable development, this paper constructs an index system for the level of sustainable agricultural development.

The design and construction process of the evaluation index system needs to focus on the following six principles: purposefulness, completeness, operability, independence, saliency, and dynamism. Independence requires each indicator to have a clear connotation and be as independent as possible. Indicators at the same level should not overlap, cross, cause and effect, or contradict each other as much as possible. Upper and lower-level indicators maintain a top-down subordinate relationship. The existence of mutual feedback and interdependence between indicator sets and among indicators within the indicator set should be avoided. At the same time, maintain good independence. The article takes the “economic system sustainable level,” “environmental system sustainable level,” “social system sustainable level,” “resource system sustainable level,” and “population system sustainable level” as the primary indicators. It ensures the independence of the primary indicators. It is also a complex, comprehensive evaluation indicator. There may be interdependence and feedback relationships between indicators to ensure the indicator system’s purposefulness, completeness, and operability. Therefore, previous literature was combined to construct agricultural sustainable development indicators for secondary indicators (Jiao^32^; Ding et al.^33^; Miao et al.^34^). This article uses the VIF test method to test multicollinearity and eliminate collinearity indicators. Meanwhile, considering the feasibility of indicators and the availability of data, a sustainable agricultural development indicator system is constructed as shown in Table 1:

**Table 1:** Index system of agricultural sustainable development level.

Primary indicators	Secondary indicators	Computing method	Unit	Attribute
Sustainability of the economic system	Rural per capita agricultural GDP	Primary industry GDP/rural population	Ten-thousand-yuan	+
	Per capita disposable income of rural residents	——	Yuan	+
Sustainability of environmental systems	Forest cover	Forest area/total area	%	+
	Damage from natural disasters	Affected area/total area	%	−
	Pesticide intensity	Total pesticide use/total cultivated land area	Ton/mu	−
	Strength of agricultural plastic film	Agricultural plastic film use/total cultivated land area	Square meters/mu	−
	Fertilizer use intensity	Total fertilizer use/total cultivated land area	Ton/mu	−
Resource system sustainability	Grain production per capita	Total grain production/total population	kilogram	+
	Level of Agricultural Mechanization	Total power of agricultural machinery/total agrarian output value	——	+
	Effective irrigation area	Irrigation area/total cultivated land area	%	+
	Land yield	Agricultural output value/crop planting area	——	+
Sustainability of social systems	Fiscal expenditure on supporting agriculture	——	Ten-thousand-yuan	+
	Per capita electricity consumption in rural areas	Total rural electricity consumption/total rural population	Watts/person	+
	Per capita consumption expenditure of rural residents	——	Yuan	+
Population system sustainability	Natural population growth rate	(births deaths)/average total population in the same period	Per thousand	+
	Average years of education in rural areas	Total years of rural education/total rural population	Year	+
	Rural ageing	Rural population over 65/total rural population	%	−

Positive indicators refer to indicators with higher values indicating a higher level of sustainable agricultural development; negative indicators refer to indicators that suggest that the smaller the indicator value, the higher the level of sustainable agricultural development.

#### Data sources and calculation method

The data on agriculturally sustainable development level indicators come from the China Rural Statistical Yearbook, China Statistical Yearbook, China Agricultural statistical yearbook, China Urban Statistical Yearbook, and provincial and Municipal Statistical Yearbooks over the years. Some indicators are obtained by estimation or calculation, and the specific calculation method is shown in Table 1. In addition, some missing data are supplemented by linear interpolations. In addition, considering the particularity and data availability of Tibet, Hong Kong Special Administrative Region, Macao Special Administrative Region, and Taiwan Province, the data of these four regions were excluded from the calculation process. Finally, 30 provincial panel data from 2006 to 2020 are obtained to calculate the level of sustainable agricultural development in various regions of China.

Accurately and efficiently measuring the level of sustainable agricultural development in China is the basis for studying its relationship with the degree of land transfer. The existing literature mainly uses factor analysis and data envelopment analysis to calculate the level of sustainable development in agriculture. Data envelopment analysis measures production efficiency from the perspectives of input and output, and its results also include slack variables for calculating inefficiency with relatively small errors. But, the degree of relationship between input variables and output variables was not considered. Therefore, this article draws on the calculation method of Zhang et al.^35^ for the sustainable development of agriculture in China and selects the entropy weight method, an objective weighting method. The weight of each indicator is determined based on the degree of variation of its values, which is more scientific and practical.

### Analysis of calculation results of agricultural sustainable development level

The above has given the calculation method and index system of agricultural sustainable development level and the data source. After calculation, this paper obtains the sustainable development level of agriculture in 30 regions of China from 2006 to 2020.

Table 2 shows that, on the whole, China’s agricultural sustainable development level shows two characteristics: first, the overall level of sustainable agricultural development shows an upward trend. The level of sustainable agricultural development in all regions in 2020 is higher than in 2006. The average growth rate in Shanghai was 13.36%. Among them, the average growth rate of agricultural sustainable development level in 11 regions is more than 5%. The five regions with the most significant average growth rate are Shanghai, Hainan Province, Fujian Province, Beijing, and Hubei Province, which are 13.36%, 7.69%, 7.45%, 7.25%, and 7.22%, respectively. Table 2 shows that except for Hubei Province, the other four regions are nongrain-producing areas, especially Shanghai and Beijing. The high level of economic development drives agriculture’s green and healthy development. Hainan, Fujian, and other regions started agricultural development late and lagged behind the “big army” considerably. However, driven by the sustainable development of agriculture in China and the acceleration of the inflow of agricultural production factors, there has been relatively rapid growth. Second, the current level of sustainable agricultural development in China is still low. The five regions with the lowest level of sustainable agricultural development in 2020 are Gansu Province, Shanxi Province, Shandong Province, Guangxi Province, and Anhui Province, which are 0.1547, 0.1944, 0.198, 0.2108, and 0.211 respectively. Shandong and Anhui are the main grain-producing areas, and other provinces are also central agricultural production provinces. Still, the level of sustainable agricultural development lags behind other regions.

**Table 2:** The sustainable development level of agriculture in 30 regions of China from 2006 to 2020.

Year	2006	2007	2008	2009	2010	2011	2012	2013	2014	2015	2016	2017	2018	2019	2020
Anhui	0.126	0.130	0.136	0.145	0.142	0.147	0.155	0.159	0.167	0.168	0.177	0.186	0.191	0.198	0.211
Beijing	0.141	0.153	0.164	0.175	0.182	0.184	0.188	0.197	0.203	0.234	0.241	0.260	0.272	0.285	0.285
Fujian	0.133	0.142	0.155	0.159	0.164	0.180	0.187	0.203	0.208	0.216	0.234	0.243	0.258	0.272	0.273
Gansu	0.108	0.098	0.106	0.108	0.107	0.106	0.115	0.111	0.126	0.133	0.125	0.134	0.137	0.151	0.155
Guangdong	0.141	0.152	0.156	0.162	0.167	0.180	0.187	0.186	0.196	0.206	0.208	0.225	0.240	0.245	0.255
Guangxi	0.136	0.138	0.133	0.147	0.145	0.157	0.153	0.171	0.177	0.178	0.181	0.185	0.202	0.205	0.211
Guizhou	0.162	0.165	0.150	0.167	0.173	0.166	0.170	0.177	0.191	0.189	0.204	0.220	0.225	0.251	0.283
Hainan	0.119	0.110	0.118	0.126	0.136	0.130	0.160	0.172	0.172	0.184	0.184	0.220	0.221	0.237	0.248
Hebei	0.165	0.162	0.162	0.171	0.176	0.170	0.179	0.191	0.186	0.201	0.195	0.198	0.209	0.215	0.222
Henan	0.139	0.140	0.136	0.149	0.138	0.153	0.163	0.167	0.168	0.168	0.182	0.177	0.189	0.198	0.213
Heilongjiang	0.198	0.179	0.208	0.208	0.214	0.222	0.230	0.231	0.254	0.253	0.264	0.279	0.283	0.298	0.348
Hubei	0.116	0.111	0.127	0.126	0.142	0.157	0.166	0.172	0.172	0.194	0.185	0.200	0.213	0.232	0.233
Hunan	0.143	0.155	0.158	0.160	0.166	0.177	0.168	0.196	0.195	0.193	0.204	0.204	0.209	0.237	0.230
Jilin	0.155	0.169	0.157	0.163	0.186	0.197	0.198	0.193	0.213	0.206	0.220	0.228	0.240	0.246	0.259
Jiangsu	0.152	0.164	0.161	0.175	0.192	0.188	0.228	0.229	0.230	0.249	0.251	0.260	0.293	0.285	0.294
Jiangxi	0.181	0.158	0.170	0.193	0.197	0.198	0.195	0.197	0.191	0.199	0.204	0.225	0.225	0.232	0.233
Liaoning	0.163	0.146	0.164	0.167	0.162	0.207	0.199	0.198	0.202	0.205	0.206	0.240	0.213	0.222	0.220
Inner Mongolia	0.180	0.192	0.210	0.204	0.209	0.202	0.217	0.214	0.220	0.223	0.243	0.240	0.257	0.259	0.298
Ningxia	0.208	0.203	0.202	0.188	0.219	0.203	0.196	0.208	0.201	0.209	0.228	0.212	0.231	0.223	0.268
Qinghai	0.215	0.237	0.228	0.202	0.176	0.188	0.181	0.178	0.185	0.199	0.197	0.197	0.197	0.246	0.283
Shandong	0.153	0.161	0.145	0.181	0.168	0.161	0.184	0.169	0.181	0.222	0.192	0.205	0.195	0.237	0.198
Shanxi	0.187	0.178	0.181	0.149	0.171	0.161	0.169	0.170	0.188	0.186	0.180	0.161	0.196	0.197	0.194
Shaanxi	0.190	0.169	0.201	0.184	0.176	0.201	0.185	0.191	0.237	0.206	0.214	0.207	0.251	0.215	0.238
Shanghai	0.186	0.197	0.182	0.216	0.209	0.223	0.235	0.433	0.437	0.413	0.423	0.465	0.498	0.516	0.533
Sichuan	0.138	0.162	0.158	0.146	0.171	0.155	0.162	0.208	0.184	0.194	0.194	0.236	0.198	0.224	0.257
Tianjin	0.196	0.171	0.193	0.185	0.187	0.205	0.225	0.228	0.245	0.230	0.261	0.265	0.252	0.265	0.280
Xinjiang	0.146	0.168	0.152	0.161	0.179	0.203	0.198	0.226	0.179	0.224	0.232	0.221	0.235	0.247	0.233
Yunnan	0.158	0.143	0.166	0.147	0.150	0.192	0.169	0.180	0.172	0.219	0.161	0.197	0.223	0.241	0.228
Zhejiang	0.218	0.195	0.201	0.224	0.260	0.257	0.272	0.237	0.292	0.306	0.301	0.318	0.331	0.314	0.348
Chongqing	0.123	0.152	0.131	0.145	0.185	0.166	0.180	0.171	0.224	0.169	0.198	0.233	0.247	0.237	0.227

The sustainable development of agriculture in China is slow and has many constraints. At the same time, regional growth is uneven, the level of sustainable agricultural development varies significantly between regions, the level of sustainable agricultural development in more developed cities remains at a high level, and the level of sustainable agricultural development in underdeveloped areas, especially in major grain-producing regions, remains at a low level.

## Empirical study on the impact of land process policy on the level of sustainable agricultural development

### Model construction and variable description

With the gradual digestion of the enthusiasm for agricultural production released by the household contract responsibility system, China has gradually shifted from a “small peasant economy” to large-scale agricultural production. In this process, transferring rural land management rights has become the key to further improving China’s rural land system and promoting sustainable agricultural development. Zhongfa [2010] No.1, issued by the Chinese government in 2010, clearly stated that “we will promote the reform of the rural land management system in an orderly manner and accelerate the registration and certification of rural collective land ownership, homestead use rights, and collective construction land use rights.” With the gradual improvement of the relevant land transfer system, the interests of both sides of the land transfer have been guaranteed to a large extent. After 2010, the scale of land transfer in China increased substantially, and there is heterogeneity in different regions and whether they are the prominent grain-producing areas. Therefore, this paper uses the DID method to conduct empirical research to reduce the endogenous problems caused by selective bias and reverse causality (Beck et al.^36^). When the sample size is small, traditional statistical techniques may lead to incorrect inference due to autocorrelation. Based on previous research, this article uses Bootstrap sampling to increase sample size and correct the bias of small sample double difference models (Bertrand and Mullianathan^37^). Bootstrap sampling is a commonly used statistical method. It constructs multiple similar resampled datasets by extracting samples with replacements from the original dataset. Thus simulating random variability and conducting statistical analysis on each resampled dataset.

However, the explanatory variable land transfer scale is not a binary virtual variable. Here, drawing on the research methods of Nunn and Qian^38^, we use the continuous DID method to estimate the impact of land transfer policy on sustainable agricultural development and use continuous variables to distinguish the treatment group and the control group. This article takes regions with land transfer scales more significant than the average as the treatment group. At the same time, areas with lower than average land transfer scale will be considered control groups. This article uses the agricultural sustainable development level calculated above as a variable to measure the effectiveness of policies. 2010 is the implementation time of the policy. Then, N control and M experimental groups were randomly and independently selected from the sample. A random algorithm determines the number of samples. Finally, a continuous double difference model regresses the extracted sample data. See Eq. (1) for the specific model setting:

1 $${ASD}_{i,t}=\alpha +{\beta }_{0}{LT}_{i,t}\times {P}_{t}+{\beta }_{1}{X}_{i,t}+{\mu }_{i}+{\theta }_{t}+{\varepsilon }_{i,t}$$

Among them,${ASD}_{i,t}$ is the explained variable, that is, the level of sustainable agricultural development in the t-Year and i-region,${LT}_{i,t}\times {P}_{t}$ is the explanatory variable, ${LT}_{i,t}$ is the continuous variable land transfer scale, ${P}_{t}$ is the virtual variable at the policy time point, when ${\text{t}}\ge 2010$,${P}_{t}$=1, when ${\text{t}}<2010$,${P}_{t}$=0; ${X}_{i,t}$ are control variables, they are industrial structure (${\text{is}}$), urbanization level (${\text{ude}}$), energy structure (${\text{eng}}$), and grain economy ratio (${\text{fsc}}$); ${\mu }_{i}$ is the fixed-year effect; ${\theta }_{t}$ is individual fixation; ${\varepsilon }_{i,t}$ is a random error term.

1. Explanatory variable: The effect of land transfer policy, expressed by the intersection of the virtual variable of policy time point and land transfer scale (${{\text{LT}}}_{{\text{i}},{\text{t}}}\times {{\text{P}}}_{{\text{t}}}$), the cultivated land area in different regions is different, and only the transfer area representing the degree of land transfer is not comprehensive enough. Hence, this paper takes the ratio of land transfer area to total cultivated land area as the scale of land transfer (${\text{LT}}$).
2. Explained variable: Agricultural sustainable development water (${\text{ASD}}$), which involves agricultural economy, rural resources, and other aspects. In the previous part of this paper, the sustainable development level of regional agriculture in China from 2006 to 2020 is estimated. The calculation results are shown in Table 2, which will not be repeated here.
3. Control variables: The control variables selected in this paper are as follows: industrial structure ($is$) reflects the process of transformation of regional industrial structure from a low level to a high level, which is expressed in this paper by (added value of tertiary industry + added value of secondary industry)/added value of primary sector; The level of urbanization ($urb$) reflects the progress of urban civilization and the gap between urban and rural development. This paper uses the ratio of the permanent urban population to the total permanent population of the region to measure. Energy structure ($ene$) is an essential part of energy system engineering research, which directly affects the final energy consumption mode of various departments of the national economy and reflects people’s living standards. This paper uses the ratio of regional coal consumption to total energy consumption. Agricultural structure ($fsc$) demonstrates the production structure of farm products, expressed as the ratio of grain planting area to the entire planting area of cash crops. The higher ratio indicates that the development of the agricultural economy in this region depends more on grain production.
4. Instrumental variable: the continuous dual difference method may have a biased and inconsistent estimation coefficient, so in the following, we try to alleviate the possible endogenous problem through the instrumental variable method. We use limited agricultural production capacity (*apc*) and employment personnel in agricultural and urban units (*eau*) as a tool variable. The farm production capacity calculation formula is the agricultural added value/total added value of agriculture, forestry, animal husbandry, and fishery.

The explanatory variables of the continuous DID model have been calculated previously. The data relating to the scale of land transfer and the cultivated land area contracted by households are from the national rural economic statistics and the annual report of China’s rural management statistics, which are calculated. The control variables come from the China Statistical Yearbook, China Urban Statistical Yearbook, China Energy Yearbook, and China Economic and social development statistical database from 2006 to 2020. Some indicators, such as industrial structure and urbanization level, are calculated according to the statistical data. The descriptive statistics of the main variables are shown in Table 3.

**Table 3:** Descriptive statistical analysis.

	Average value	Standard deviation	Min	Max
$LT$	2.368	3.032	0.110	33.660
$ASD$	0.200	0.056	0.098	0.533
$is$	23.36	51.06	2.29	372.67
$ude$	0.553	0.137	0.270	0.940
$eng$	0.738	0.143	0.039	0.940
$fsc$	2.924	3.837	0.490	33.190
$g$	0.525	0.085	0.338	0.746

### Empirical results

#### Analysis of benchmark regression results

Before baseline regression, we used the variance inflation factor test to rule out the possibility of multiple collinearities of explanatory variables. Table 4 shows the benchmark regression results of the impact of land transfer policies on sustainable agricultural development. Model (1) and model (3) are the benchmark regression results of common standard error and robust standard error, respectively, and model (2) and model (4) are the benchmark regression results of common standard error and robust standard error after adding control variables.

**Table 4:** Benchmark regression results.

	Common standard error		Robust standard error	
	(1)	(2)	(3)	(4)
${{\text{LT}}}_{{\text{i}},{\text{t}}}\times {{\text{P}}}_{{\text{t}}}$	0.0033*** (14.22)	0.0015*** (6.65)	0.0032*** (8.24)	0.0006* (1.78)
$is$		0.0009*** (14.27)		0.0011*** (4.27)
$urb$		0.2032*** (7.55)		0.3384*** (7.27)
$eng$		0.0497* (1.86)		0.0532 (0.71)
$fsc$		0.0026*** (3.47)		0.0024*** (3.86)
Cons	0.1620*** (21.38)	0.0047 (0.17)	0.1619*** (36.08)	− 0.0660 (− 0.99)
Control variable	No	Yes	No	Yes
Time effect	Yes	Yes	Yes	Yes
Provincial effect	Yes	Yes	Yes	Yes
R-squared	0.3271	0.6594	0.3271	0.6759
N	450	450	450	450

The values in parentheses are for t value. ***, **, and * represent the significance levels of 1%, 5%, and 10% for the regression results, respectively. Tables 5, 6, and 7 are the same.

According to the regression results in Table 4, no matter which model, the land transfer policy will positively impact sustainable agricultural development. However, to avoid the inconsistency of results caused by heteroscedasticity, it is more accurate to use robust standard error regression with control variables. The benchmark regression results of model (4) show that the coefficient of explanatory variables (${{\text{LT}}}_{{\text{i}},{\text{t}}}\times {{\text{P}}}_{{\text{t}}}$) is significantly positive at the 10% level, with a coefficient of 0.0006. It shows that the land transfer policy can improve sustainable agricultural development. The land transfer system has been further enhanced to protect the interests of both sides of the transfer, the scale of land transfer has increased substantially, the process of large-scale agricultural production has been accelerated, and the rural surplus labor force can be released. Advanced production technologies and concepts can be introduced to promote the sustainable development of agriculture.

In model (4), the control variables industrial structure, urbanization level, and agricultural structure positively impact sustainable agricultural development, with coefficients of 0.0011, 0.3384, and 0.0024, respectively, and all of them passed the significance test of 1%. The sustainable development of agriculture is affected by many factors, such as upgrading industrial structure, which reduces the proportion of agricultural added value in total GDP. Still, the rapid development of the secondary and tertiary industries will feed back agriculture, promote the efficiency of agricultural production, and promote the low-carbon development of agriculture.

### Effectiveness analysis of the DID method

The previous paper used the continuous DID regression model to verify that the land transfer policy will promote sustainable agricultural development. One of the most critical assumptions of the dual difference model is to meet the parallel trend assumption. That is, before the implementation of land policies, the level of sustainable agricultural development in different regions had a standard change trend. Next, this paper uses the parallel trend hypothesis test and the balance test to analyze the effectiveness of the did method.

#### Parallel trend hypothesis test

Considering that the implementation of land transfer policy lags and the implementation effect also needs to be digested year by year, this paper constructs a model to test the parallel trend hypothesis, and the test model is shown in formula (2).

2 $${ASD}_{i,t}=\alpha +\prod_{t\ge -4}^{10}{\alpha }_{t}{Q}_{t}+{\beta }_{0}{X}_{i,t}+{\mu }_{i}+{\theta }_{t}+{\varepsilon }_{i,t}$$

Among them, ${{\text{Q}}}_{{\text{t}}}$ is the implementation year of the land transfer policy. Because the benchmark regression model in this paper selects continuous DID and the explanatory variable is not virtual, the explanatory variable needs to be converted into a virtual variable when conducting the balanced trend test. Here, ${{\text{Q}}}_{0}$ is set as the virtual variable for the implementation of the land policy in 2010,${{\text{Q}}}_{-1}$ is the year before the implementation of the policy, and ${{\text{Q}}}_{1}$ is the year after the implementation of the policy. ${{\text{X}}}_{{\text{i}},{\text{t}}}$ is control variables, they are industrial structure (${\text{is}}$), urbanization level (${\text{ude}}$), energy structure (${\text{eng}}$), and grain economy ratio (${\text{fsc}}$); ${\upmu }_{{\text{i}}}$ is the fixed-year effect; ${\uptheta }_{{\text{t}}}$ is individual fixation; ${\upvarepsilon }_{{\text{i}},{\text{t}}}$ is a random error term.

To avoid multicollinearity, we take the year before the implementation of the policy as the benchmark group and lose 2009 in the regression to estimate the regression coefficient from 2006 to 2020, that is, {β_−3_, β_−2_, β_0_, β_1_, β_2_, β_2_, β_3_, β_4_, β_5_, β_6_, β_7_, β_8_, β_9_, β_10_}, then draw the value and confidence interval of the regression coefficient in the chart. The specific steps are as follows: first, the regression results (mainly including regression coefficient and standard error) are derived, and then the regression results are sorted out, and then the confidence intervals of each interaction coefficient are calculated. Finally, Stata software draws the value and confidence interval of the regression coefficient. Figure 3 shows the results of the parallel trend hypothesis test.

**Figure 2:**
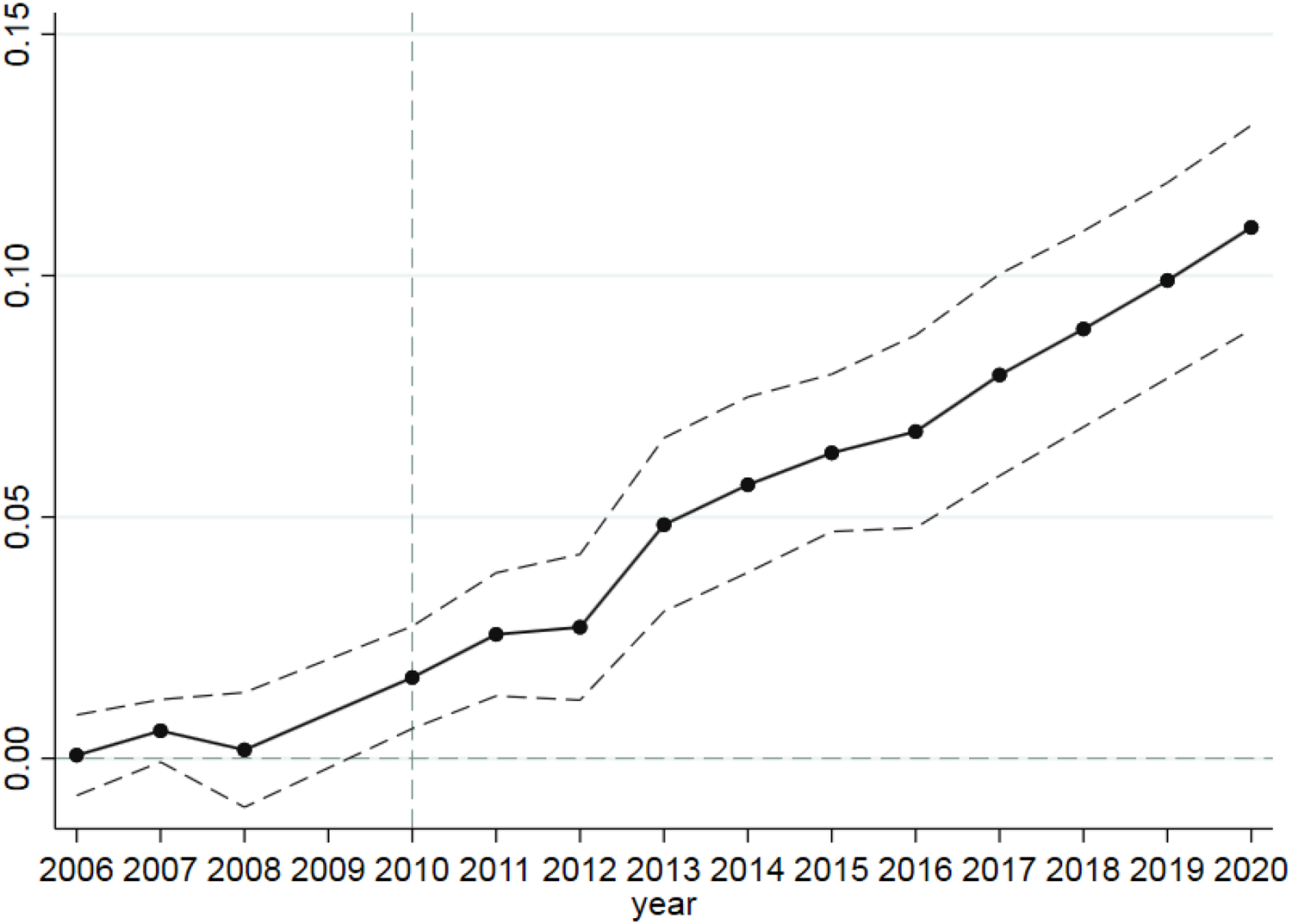
Parallel trend hypothesis test results.

As can be seen from Fig. 2, before 2010, the coefficient of the interaction term was basically around 0, especially when the 95% confidence interval included 0. After 2010, it was apparent that the regression coefficient was more significant than 0, and the 95% confidence interval was above 0. It shows that before the implementation of the land transfer policy, the degree of land transfer and the level of sustainable agricultural development did not show a heterogeneous time trend, and the significance is not vital. That is to say, the relationship between the degree of land transfer and sustainable agricultural development is constant over time. After implementing the policy, the impact of the land transfer policy on sustainable agricultural development is significantly positive. The effect of land transfer policy on agricultural sustainable development gradually increases with the implementation, which shows that the impact of land transfer policy on agricultural sustainable development is delayed and sustained. In the early stage of the implementation of the land transfer policy, The effect of the expansion of the land transfer scale is mainly reflected in the increase of agricultural production efficiency, which is only one aspect of sustainable agricultural development. To sum up, the test results of the equilibrium trend hypothesis given in Fig. 2 prove that the continuous DID regression model meets the equilibrium trend hypothesis, and the implementation of the land transfer policy has sustainability and lag, which also helps to prove the benchmark regression results given in Table 4 to a certain extent.

#### Balance test

**Figure 3:**
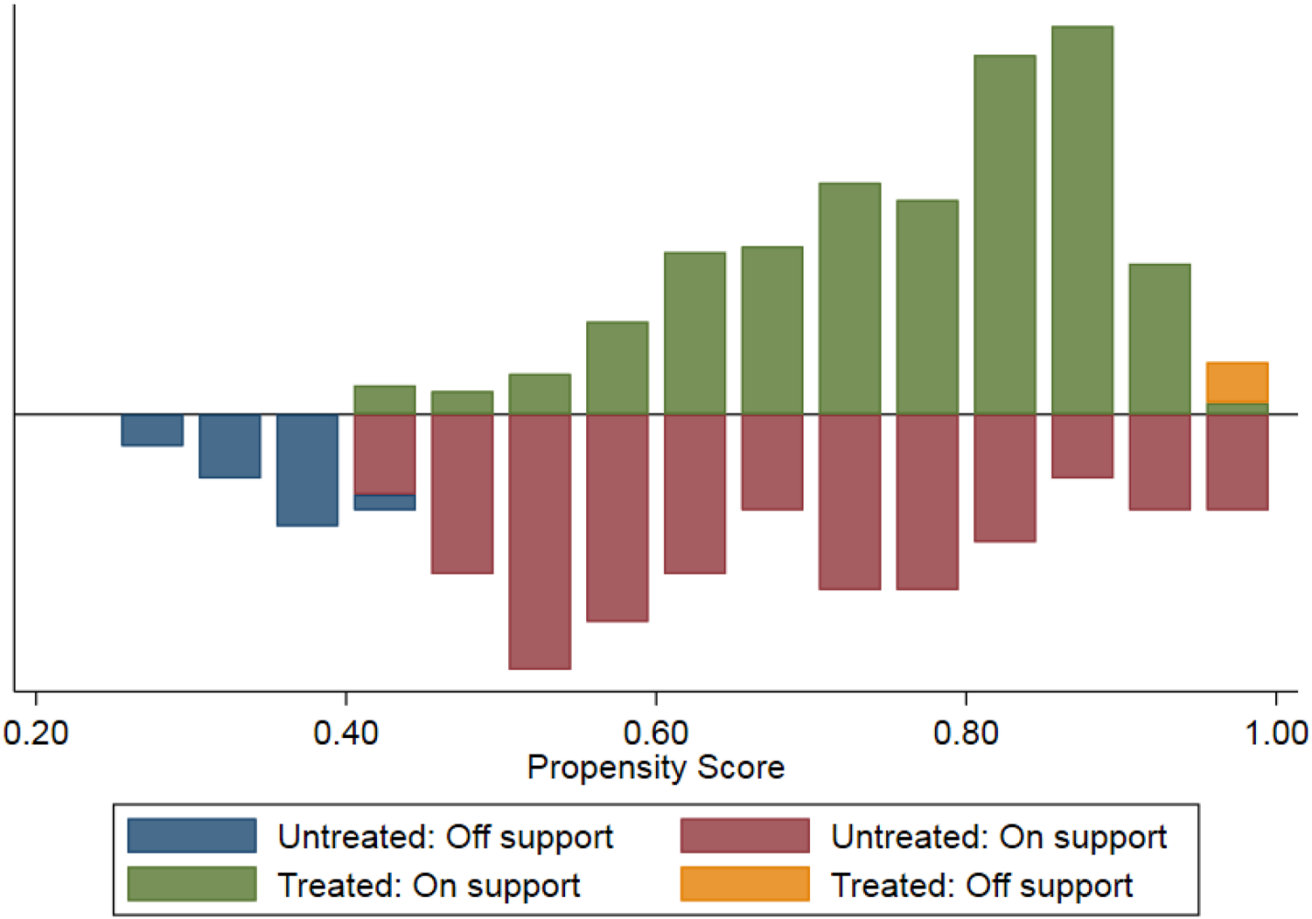
The standard range of propensity scores.

The above verifies that the model meets the equilibrium trend hypothesis, but when using the continuous did model for empirical analysis, there may also be a self-selection problem of land transfer, that is, whether the land contractor carries out the land transfer is not strictly exogenous, but also affected by family labor structure, non-farm income, their education, region, children’s occupation and other factors, which may lead to self-selection bias. As a result, the estimated results are biased. Therefore, to reduce the bias of DID estimation, this paper further uses the propensity score matching method (PSM) to correct the possible selectivity bias and conduct a balance test. Figure 3 shows the distribution of propensity scores in the experimental and control groups.

As can be seen from Fig. 3, after matching, the standardization deviation of almost all covariables has decreased significantly, indicating that the standard deviations of explanatory variables are less than 10%, and the t-test results are not significant, so it has passed the joint test. The study found that after pairing, there was no significant difference in each index between the experimental froup and the control groups. After the matching, the absolute value of the standard deviation of each variable has decreased significantly, and the distribution of each variable between the experimental group and the control group tends to be balanced, indicating that the matching quality is relatively high and can meet the equilibrium hypothesis. As shown in Fig. 3, all observations meet the standard of tendency score, and only a few samples are lost in pairing. The results showed that after treatment, the propensity scores of both the experimental and control groups improved, indicating that the matching quality of the two methods was higher. The study found that before the implementation of the agricultural land transfer system and before the implementation of the two stages, the data of each stage have gone through a balanced test, indicating that the method proposed in this paper is feasible.

#### Endogenous test

The above analysis only uses the continuous DID regression model, which can not avoid the potential endogenous problems in the model; there may be missing variables, and the previous empirical results may not be accurate enough. Although this paper considers the complex factors affecting sustainable agricultural development and the non-observational factors that are variable or immutable over time in calculating and demonstrating sustainable agricultural development, the factors affecting the level of sustainable agricultural development are complex. It may still be challenging to characterize and measure factors, such as the differences in institutional policies applicable to local governments. The control variables can not completely effectively prevent the existence of missing variables. Therefore, to further verify the reliability of the benchmark regression results, this paper selects the ratio of agricultural added value to the total added value of agriculture, forestry, animal husbandry, and fishery, and employment personnel in agricultural and urban units as the instrumental variable of the land transfer scale. The reason is that in terms of correlation, in areas with the same level of urbanization and agricultural development, the higher the proportion of narrow agricultural added value, the more Farmers in the region are willing to transfer land. At the same time, non-rural residents are more inclined to move to land for large-scale production. Land transfer: the larger the transfer scale, the more agricultural and urban units need to employ people. Considering that instrumental variables need to meet the externality, although the purchase price of grain is affected by market supply and demand, the grain price regulation policy implemented by the Chinese government to protect the interests of farmers and ensure the supply of grain market implements the minimum purchase price in the prominent grain producing areas when necessary, which provides that the cost of agricultural products is not different in different regions. Hence, the proportion of agricultural added value in a region depends on the local natural factor endowment. It will not directly impact sustainable agricultural development. There is also no correlation between the employment of agricultural and urban units and the level of sustainable agricultural development. So, it meets the exogenous requirements of instrumental variables. The instrumental variables’ data sources and descriptive statistical analysis have been given above. Then, we use the two-stage least square method to test the endogenous nature of the model. Table 5 shows the estimated results of the instrumental variables, including the first-order and second-order regression results.

**Table 5:** Two-stage regression results using instrumental variables.

	(1)	(2)
${\text{DID}}$	0.0101** (2.34)	0.0131*** (3.50)
Cons	0.1779*** (18.40)	0.1592*** (17.15)
Control variable	Yes	Yes
Time effect	No	Yes
Provincial effect	No	Yes
R^2^	0.1778	0.4429
Overidentification test results	*p* = 0.5916	*p* = 0.8108
N	450	450

According to the results of Table 5, in the weak tool test of model (1) and model (2), the p-value of F statistics of kleibergen Paaprk LM is significant at 1%. The F statistics are all above 10, which exceeds the threshold of the weak discriminant test. In the over-recognition test, the p-values of model (1) and model (2) were 0.5916 and 0.8108, respectively. Therefore, accept the original assumption. It indicates that the narrow agricultural production capacity and the exogenous employment of agricultural and urban units are unrelated to the disturbance term. Therefore, it is reasonable for this article to choose agrarian production capacity agricultural, and urban unit employment as instrumental variables for land transfer scale.

Furthermore, the estimated coefficients for narrow agricultural production capacity and agricultural urban unit employment in the first stage are 0.0133 and 5.490, respectively. And it passed the significance test at the 5% level. It also indicates that there is a correlation between instrumental variables and explanatory variables. The estimated coefficients of the explanatory variables for model (1) and model (2) in the second stage estimation results are 0.0101 and 0.0131, respectively, with positive values. And they passed the significance level tests of 5% and 1%, respectively. Considering the endogenous, the land transfer policy will still promote the sustainable development of agriculture. Compared with the benchmark regression, the results of the instrumental variable regression have not changed substantially, and the instrumental variable regression further proves the conclusion of the benchmark regression.

### Robustness test

#### Placebo test

The above empirical conclusions show that this method has successfully overcome the assumption and endogenesis of parallel tendency. However, this does not fully explain how the trend between the two control groups will change after different policy intervention times. And how other related policies or random factors will change. In response, we have to do some placebo tests. The central idea of the placebo trial is to estimate a hypothetical treatment group or hypothetical treatment cycle. Suppose the regression results of the estimates under the fictitious treatment group or the fictitious policy time are insignificant. In that case, it can be said that the original estimates have passed the placebo test, and the land transfer policy can promote agriculture’s sustainable development. Next, we learn from the practices of Shi^39^ and Li et al.^40^ scholars to fabricate the policy time for the placebo test, and Table 6 gives the results of the placebo test.

**Table 6:** Placebo test results.

	Policy time: 2009		Policy time: 2018	
	Common standard error (1)	Robust standard error (2)	Common standard error (3)	Robust standard error (4)
${{\text{LT}}}_{{\text{i}},{\text{t}}}\times {{\text{P}}}_{{\text{t}}}$	0.0014 (0.89)	− 0.0030 (− 0.74)	0.0009 (0.73)	− 0.0018 (− 1.34)
Cons	− 0.0552 (− 1.16)	− 0.0826 (− 1.42)	− 0.0514 (− 1.21)	− 0.0873 (− 1.41)
Control variable	Yes	Yes	Yes	Yes
Time effect	Yes	Yes	Yes	Yes
Provincial effect	Yes	Yes	Yes	Yes
R-squared	0.6567	0.6796	0.6581	0.6776
N	450	450	450	450

Placebo test ①: the essence of the placebo test has been analyzed in detail above to prove no significant difference in green innovation behavior between the experimental and control groups before the policy shock. Therefore, the year before the policy implementation is set as the policy implementation year of the treatment group. The same fixed time and province effects were used, using the same control variables as the baseline regression. And then the continuous DID model regression is carried out. The results show that whether it is the general standard error regression result of the model (1) or the robust standard error regression result of the model (2), the coefficient of the core explanatory variable land transfer policy has not passed the 10% significance level test. The estimated results are consistent with the expected results. It can be excluded that the trend changes of the treatment and control groups after the land transfer policy are affected by other policies or random factors. The coefficient of explanatory variables was not significant and passed the placebo test.

Placebo test ②: To further eliminate other policy interference, the time point of the land transfer policy will be changed to 2018. The same fixed time and province effects were used, using the same control variables as the baseline regression. Then, the continuous DID model regression is carried out. The results show that the coefficient of land transfer policy, the core explanatory variable, has not passed the 10% significance level test, and the estimated results are consistent with the expected results. Excluding other policy disturbances, the benchmark estimated results are credible and have passed the placebo test.

#### Robustness test

Sustainable agricultural development is affected by many factors, such as changes in rural population structure and economic development stage, which will fluctuate. Therefore, to ensure the rigor of the demonstration, this paper tests the robustness of the continuous did model again and makes a simple analysis by deleting the data of the policy year and controlling variables lagging behind one period. The results of the robustness test are shown in Table 7.

**Table 7:** robustness test results.

	Delete 2010 data		Control variables lag by one period	
	Common standard error (1)	Robust standard error (2)	Common standard error (3)	Robust standard error (4)
${{\text{LT}}}_{{\text{i}},{\text{t}}}\times {{\text{P}}}_{{\text{t}}}$	0.0020** (2.49)	0.0022** (2.50)	0.002** (2.55)	0.0026*** (2.97)
Cons	− 0.0524* (− 1.87)	− 0.0804*** (− 2.83)	− 0.0392 (− 1.42)	− 0.0763*** (− 2.72)
Control variable	Yes	Yes	Yes	Yes
Time effect	Yes	Yes	Yes	Yes
Provincial effect	Yes	Yes	Yes	Yes
R-squared	0.6530	0.6776	0.6317	0.6630
N	420	420	420	420

① Delete the data of the policy year. Model (1) and model (2) in Table 7 are the regression results of continuous DID common standard error and robust standard error after deleting the data in 2010, respectively. It can be seen that the regression coefficient of the land transfer policy is significantly positive, and both are significant at the 5% level, which is consistent with the benchmark regression results in Table 4. The regression results after deleting the data of the policy implementation node do not deviate much, indicating that the empirical results are stable.

② The control variable lags by one period. Table 7, model (3), and model (4) are the regression results of continuous DID common standard error and robust standard error with control variables lagging one year, respectively. It can be seen that the regression coefficient of the land transfer policy is 0.002 and 0.0026, respectively, which is significant at the 5% and 1% levels, respectively. It is also basically consistent with the benchmark regression results in Table 4. The regression results of control variables lagging behind one period do not deviate much, indicating that the empirical results are robust.

### Heterogeneity analysis

Heterogeneity analysis can help reveal potential patterns and trends in the dataset. We discover their differences and patterns by comparing data from different subpopulations or under different conditions. Then, obtain more comprehensive and accurate information. Due to other agricultural practices, cultural differences, or economic development levels, land transfer policies may impact different regions. Therefore, this article conducts heterogeneity testing. Firstly, based on geographical location, the 30 provinces are divided into the eastern, central, and western regions. Conduct slight sample random continuous double difference regression separately. The control variables are the same as those of the benchmark regression model. The results are shown in Table 8. In addition, there are a total of 13 prominent grain-producing areas in China, namely Heilongjiang, Henan, Shandong, Sichuan, Jiangsu, Hebei, Jilin, Anhui, Hunan, Hubei, Inner Mongolia, Jiangxi, and Liaoning. The prominent grain-producing areas include 100 central grain-producing counties. According to whether it is a major grain-producing area, 30 provinces are divided into two categories: main grain-producing areas and non-major grain-producing areas. And further verify heterogeneity. The regression results are shown in Table 8.

**Table 8:** Geographical Location Heterogeneity Results.

	Economic zoning		
	(1) Eastern	(2) Central	(3) Western
DID	0.0023* (1.66)	0.0043** (2.32)	− 0.0054 (− 1.66)
Intercept	− 0.1466** (− 2.33)	0.1886*** (2.87)	.0304 (0.71)
Control variable	Yes	Yes	Yes
Time effect	Yes	Yes	Yes
Provincial effect	Yes	Yes	Yes
R-squared	0.6770	0.7800	0.6034

From Table 8, it can be seen that the regression coefficients for the eastern and western regions are positive. The coefficients are 0.0023 and 0.0043, respectively. They passed the 10% and 5% significance tests, respectively. The effectiveness of land transfer policies in the central region is higher than in the eastern region. The central region usually has relatively rich agricultural and land resources.

In contrast, the eastern region is densely populated and has relatively limited land resources. Agriculture is more developed in the central area, and implementing land transfer policies makes it easier to promote the optimal allocation and development of resources. The regression results in the western region did not pass the 10% significance test. Therefore, it is impossible to determine the effectiveness of land transfer policies in the Western region. The western region usually has geographical and natural limitations such as complex terrain, numerous mountainous areas, and scarce water resources. These factors lead to significant difficulties in land use and circulation. At the same time, it is not conducive to the implementation and effectiveness of land transfer policies.

Table 9 shows that the positive effect of land transfer policies in prominent grain-producing areas on sustainable agricultural development has passed the 1% significance test. The coefficient is 0.0057. In contrast, the impact of land transfer policies in non-grain-producing areas on sustainable agricultural development cannot be entirely determined. The prominent grain-producing regions have a relatively complete agricultural industry chain and agricultural product market system. Collaborative cooperation in planting, processing, and sales can be achieved through land transfer. They were simultaneously forming a whole industrial chain. Therefore, it promotes the value enhancement of agricultural product processing and sustainable agricultural development.

**Table 9:** Results of Grain Location Heterogeneity.

	Grain zoning	
	(4) Major grain-producing area	(5) Non-major grain-producing areas
DID	0.0057*** (3.87)	0.0015 (1.49)
Intercept	0.0639*** (1.54)	− 0.04471 (− 1.23)
Control variable	Yes	Yes
Time effect	Yes	Yes
Provincial effect	Yes	Yes
R-squared	0.8044	0.6015

## Conclusions, policy recommendations, and research prospects

### Conclusions

This article establishes a measurement index system for agricultural sustainable development based on panel data from 30 regions in China from 2006 to 2020. In order to reduce endogeneity issues caused by selective bias and reverse causal relationships, this article uses the continuous double difference method (Differences in Differences) to empirically test whether land transfer policies can promote sustainable agricultural development. The conclusion is as follows:

1. The sustainable development level of agriculture in China presents two characteristics. Firstly, the overall level of sustainable agricultural development shows an upward trend. Secondly, the current level of sustainable agricultural development in China is still relatively low. Among the five regions with the lowest level of sustainable agricultural development in 2020, Shandong and Anhui are the prominent grain-producing regions, while other provinces are also significant producers of farm products. However, the level of sustainable development in agriculture lags behind other areas.
2. Land transfer policies will have a positive impact on sustainable agricultural development. The land transfer system has been further improved to ensure the interests of both parties involved. The significant increase in land transfer has accelerated the process of large-scale agricultural production. At the same time, it can release surplus rural labor, introduce advanced production technologies and concepts, and promote sustainable agricultural development.

### Policy recommendations

The above results show that land transfer policy can promote sustainable agricultural development. At this stage, China has not yet completed industrialization, and urbanization is still in an unstable population spatial structure. Therefore, this paper puts forward specific policy suggestions as follows:

1. Improve the land transfer market and protect the interests of farmers. In 2016, the Chinese government issued opinions on improving the separation of rural land ownership contracting rights and management rights, dividing rural land contracting rights into contracting and management rights, and implementing the separation of ownership, contracting rights, and management rights. China’s land transfer policy has been relatively perfect, and the land transfer market needs to be improved simultaneously. The government can strengthen the supervision of the land transfer market to ensure the fairness, fairness, and security of transactions. At the same time, a series of measures can be taken to encourage the transfer, such as providing free land information, reducing transfer fees, increasing financial support, and so on. We also need to support the development of land management entities. The government can encourage the development of land management entities and improve their management level and competitiveness through financial support and preferential taxation. At the same time, the government establishes a sound land transfer contract system to protect farmers’ land contract rights, transfer rights, and income rights, prevent the improper or unreasonable distribution of interests in land transfer, and safeguard farmers’ legitimate rights and interests.
2. Strengthen agricultural production services and increase land trusteeship services. The ultimate purpose of the land transfer policy is to improve the efficiency of agricultural production and maintain the farm production environment. Therefore, to better play the positive effect of land transfer policy and promote sustainable agricultural development, the government can strengthen agricultural technology training, scientific and technological innovation, and agricultural information construction, improve the efficiency and quality of agricultural production, and further improve the income level of farmers. In addition, the government should establish a sound land trusteeship mechanism, strengthen the supervision and services of land trusteeship, ensure the protection and effective use of land resources, and improve farmers’ land income. Meanwhile,
3. Promote the adjustment of agricultural structure and promote agricultural technological innovation. The core of sustainable agricultural development is structural adjustment and technological innovation. The government should encourage the structural adjustment of agriculture and realize the upgrading and transformation of agriculture by reforming the land system, strengthening rural land transfer, optimizing the structure of cultivated land, and promoting efficient and water-saving agriculture. Technological innovation requires a lot of investment. A small peasant economy dominates China’s agriculture and lacks large farmers to develop new technologies. Therefore, the government should increase support for agricultural technological innovation, strengthen the training of scientific and technical talents and investment in scientific and technical research and development, encourage farmers to improve agricultural production efficiency and quality through scientific and technological innovation and reduce environmental pollution.
4. Improve the efficiency of rural resource utilization and improve the environmental protection system. First, measures should be taken to promote the efficiency of agricultural production and the efficiency of resource utilization. For example, we should develop organic agriculture, Nongjiale tourism, and circular agriculture, reduce the use of non-renewable resources such as pesticides, fertilizers, and water, and improve the efficiency of resource recovery and reuse. Secondly, we should establish a sound ecological protection system and supervision mechanism, protect natural resources such as water, soil, gas, and light needed for agricultural production, reduce the impact of agricultural production on the environment, and achieve sustainable agricultural development. Thirdly, we should increase policy guidance and economic support for sustainable agricultural development, encourage farmers and enterprises to participate in sustainable agricultural development through fiscal and tax support and financial support and convey the concept of sustainable agricultural development to consumers.

### Research outlook

Currently, the constraints on water and soil resources in China are tightening daily, and the degradation of the agricultural ecosystem is apparent. It is urgent to formulate relevant policies to promote sustainable agricultural development. In this context, taking sustainable agricultural development as the research object, it is significant to study the impact of land transfer policy on sustainable agricultural development. However, there are still some defects in this paper. First, in calculating sustainable agricultural development, this paper only considers quantifiable indicators and obtains relevant data from the statistical yearbook and the official website of the Bureau of Statistics. Non-quantifiable indicators, such as environmental carrying capacity, sustainable development capacity of resources, barren degree of land, and so on, are not included in the index system. In this way, the index system of sustainable agricultural development is not perfect and comprehensive, so the following research will focus on the indicators that are difficult to quantify, find effective ways to incorporate them into the index system, and further improve the calculation and study of sustainable agricultural development; Secondly, due to the limited availability of data and the lack of suitable methods, this article did not conduct an extended research on the lag and continuity of land transfer policies in promoting sustainable agricultural development. The author will continue to search for appropriate methods and data to demonstrate further whether there is a lag and continuity in the impact of land transfer policies on sustainable agricultural development.

## Data Availability

The datasets used and analyzed during the current study are available from the corresponding author upon reasonable request.
